# High Volume-Per-Dose and Low Resistivity of Cobalt Nanowires Grown by Ga^+^ Focused Ion Beam Induced Deposition

**DOI:** 10.3390/nano9121715

**Published:** 2019-12-01

**Authors:** Carlos Sanz-Martín, César Magén, José María De Teresa

**Affiliations:** 1 Instituto de Ciencia de Materiales de Aragón (ICMA), Universidad de Zaragoza-CSIC, 50009 Zaragoza, Spain; carlos10sanz@gmail.com (C.S.-M.); cmagend@unizar.es (C.M.); 2 Departamento de Física de la Materia Condensada, Universidad de Zaragoza, 50009 Zaragoza, Spain; 3 Laboratorio de Microscopías Avanzadas (LMA), Instituto de Nanociencia de Aragón (INA), Universidad de Zaragoza, 50018 Zaragoza, Spain

**Keywords:** cobalt nanowires, focused ion beam induced deposition, ion milling, high growth rate, low electrical resistivity

## Abstract

The growth of ferromagnetic nanostructures by means of focused-Ga^+^-beam-induced deposition (Ga^+^-FIBID) using the Co_2_(CO)_8_ precursor has been systematically investigated. The work aimed to obtain growth conditions allowing for the simultaneous occurrence of high growth speed, good lateral resolution, low electrical resistivity, and ferromagnetic behavior. As a first result, it has been found that the competition between deposition and milling that is produced by the Ga^+^ beam is a limiting factor. In our working conditions, with the maximum available precursor flux, the maximum deposit thickness has been found to be 65 nm. The obtained volumetric growth rate is at least 50 times higher than in the case of deposition by focused-electron-beam-induced deposition. The lateral resolution of the deposits can be as good as 50 nm while using Ga^+^-beam currents lower than 10 pA. The high metallic content of the as-grown deposits gives rise to a low electrical resistivity, within the range 20–40 µΩ·cm. Magnetic measurements confirm the ferromagnetic nature of the deposits at room temperature. In conclusion, the set of obtained results indicates that the growth of functional ferromagnetic nanostructures by Ga^+^-FIBID while using the Co_2_(CO)_8_ precursor is a viable and competitive technique when compared to related nanofabrication techniques.

## 1. Introduction

Magnetic nanostructures are under development for various applications, such as hard-disk memories [1], magnetic random access memories [2], drug delivery [3], magnetic hyperthermia [4], magnetic resonance imaging [3], neuromorphic computing [5], etc. Their controlled fabrication is key in achieving the required functionality, given that the magnetic properties are greatly affected by features, such as dimensions, topology, surface roughness, interfacing, defects, crystal structure, etc. [6,7]. Focused Electron Beam Induced Deposition (FEBID) is one of the existing techniques for the growth of magnetic nanostructures, as recently reviewed [8,9]. In this technique, a precursor material containing a magnetic element is injected in the process chamber and then dissociated by a focused electron beam. Typically, a Scanning Electron Microscope (SEM) is used for FEBID, and various scanning strategies allow for the growth of magnetic dots [10], nanowires [11], pillars and nano-helices [12,13], nanospheres [14], spin-ice structures [15], etc. FEBID is well suited to be used on unconventional substrates, such as cantilevers, leading to its application in magnetic force microscopy [16,17,18] and magnetic resonance force microscopy, in sharp contrast to other nanofabrication techniques requiring resists [19]. Other reported applications of magnetic FEBID deposits comprise nanoactuation [20], domain-wall pinning [21], nano-Hall sensors [22], nanomagnetic logic [23], superconducting-vortex pinning [24,25], etc. In addition, the capability of FEBID for the growth of complex three-dimensional (3D) magnetic structures is being explored in the rising field of 3D nanomagnetism [26].

FEBID has long suffered from two disadvantages when compared to other nanofabrication techniques: the material purity and the growth speed. In the last years, various growth strategies and post-growth purification treatments have been successfully developed to enhance the material purity of magnetic deposits that are grown by FEBID [27,28,29,30,31,32]. However, the growth speed of magnetic nanostructures by FEBID continues to be a bottleneck regarding its broader use. Existing literature on the topic indicates that the volumetric growth rate of nanostructures with the widely-used Co_2_(CO)_8_ precursor is in the range of 0.002 µm^3^/nC [33]. Low electron currents must be used to obtain magnetic structures with good lateral resolution (below 1 nA), which gives rise to long growth times. As an example, the growth of a 100 nm-thick 1 µm^2^-rectangle takes 8 min. if an electron beam current of 100 pA is used.

In the present work, we have systematically explored the possibility of using a Ga^+^ focused ion beam to grow Co magnetic nanostructures with good lateral resolution, low electrical resistance, and high growth speed by means of the Focused Ion Beam Induced Deposition (FIBID) technique. Previous work on this topic is limited to one publication on the use of He^+^-FIBID to grow high-resolution Co nanolines [34] as well as scattered results on the ferromagnetic character of some Ga^+^-FIBID Co structures [35,36,37], but without a systematic comparison with their FEBID counterparts in terms of attainable dimensions, growth speed, electrical resistivity, and magnetic behavior. As will be shown hereafter, the growth of Co nanostructures by Ga^+^-FIBID is competitive in terms of growth speed, lateral resolution, electrical resistivity, and magnetic behavior, representing a promising technique in the case that these properties need to be simultaneously optimized.

## 2. Materials and Methods

The Co nanowires (NWs) were grown by FIBID in a commercial Helios Nanolab 600 Dual Beam equipment (FEI company, Hillsboro, OR, USA), with the Ga^+^ ion source being operated at 30 kV (the highest available value, to maximize the lateral resolution of the deposits, owing to the reduction of the lenses’ aberrations with voltage) and a Gas Injection System (GIS) delivering Co_2_(CO)_8_ as gas precursor into the chamber with typical base pressure of ~3.5 × 10^−6^ mbar and GIS temperature of 29 °C. The Co_2_(CO)_8_ flux is controlled via a manual valve, which was completely open to achieve the maximum precursor flux on the growth area, leading to a chamber working pressure of ~6.5 × 10^−6^ mbar. The gas needle was set at 20–30 µm in height and 30–40 µm in lateral distance from the writing field position, to allow for the gas properly reach the substrate. The ion beam was scanned while using rectangular patterns with dimensions 10 µm × 1 nm, whose thickness depends on the selected number of passes that the beam performs. Regarding the substrate, the Co-FIBID NWs were deposited on a (100) Si wafer (with ~1 nm of native oxide layer) for the structural and chemical analyses through Scanning Electron microscopy (SEM) inspection in the Dual Beam equipment.

Transmission Electron Microscopy (TEM) carried out in-depth structural and chemical characterization of the Co-FIBID NWs. For that purpose, cross-sectional lamellae of the selected specimens were extracted from the Si wafer and then attached to TEM Cu grids in the Dual Beam equipment. High-Resolution Transmission Electron Microscopy (HRTEM) images were obtained in an FEI Titan Cube operated at 300 kV and then equipped with a Field Emission Gun (FEG) and an image Cs corrector. High-Angle Annular Dark Field (HAADF) images in Scanning Transmission Electron Microscopy (STEM) mode were obtained at 300 kV in an FEI Titan Low Base (FEI company, Hillsboro, OR, USA) that was fitted with high brightness Field Emission Gun (X-FEG), a gun monochromator, and a probe-Cs corrector. Electron Energy Loss Spectroscopy (EELS) chemical analyses were performed in the Tridiem 866 ERS Gatan Imaging Filter (GIF) (Gatan Inc., Pleasanton, CA, USA) in STEM mode in the Titan Low Base. Energy Dispersive X-Ray Spectroscopy (EDS) experiments were also undertaken in the Titan Low Base in STEM mode.

The transport properties were studied by four-point electrical microprobe measurements while using electrically conductive microprobes (Kleindiek Nanotechnik GmbH, Reutlingen, Germany) connected to Keithley 2000 multimeter (Keithley Instruments, Cleveland, OH, USA) placed out of the Helios Nanolab 650 Dual Beam chamber. On a 300 nm-SiO_2_ layer on (100) Si wafer, an optical lithography lift-off process was undertaken to obtain conductive Ti metal pads. After that, four Pt-FIBID contacts were grown between the NWs and the electrodes for subsequent electrical measurements.

MOKE experiments were carried out in longitudinal configuration in a NanoMOKE3 magnetometer (Durham Magneto Optics Ltd. company, Caxton, UK), while applying the magnetic field in the deposits plane, in sweeps of 600 Oe as the maximum value and frequency variations between 0.5 Hz and 2.0 Hz. Several loops (between 20–30) were averaged for a better signal-to-noise ratio. For this examination, arrays of eight NWs were grown on the (100) Si wafer to obtain a well-detectable MOKE signal.

## 3. Results and Discussion

### 3.1. Optimization of the Growth Parameters

The research was focused on the variation of the ion dose applied to the deposits. Such ion dose is calculated from the ion current and the deposit time. The ion beam current was selected in the pA range to both guarantee the growth of narrow structures and avoid excessive ion milling. In particular, the two lowest currents available in the equipment were selected: 1.5 pA and 9.7 pA (with the experimental values being 1.39 pA and 10.67 pA, respectively) to analyze the difference between the resulting NWs. As for the deposit time, the variation was centered on modifying the number of passes (up to several millions) that the beam scans over the pattern. Thus, the dimensions of the Co-FIBID NWs were examined as a function of this determining factor when considering this varying ion dose.

**Figure 1:**
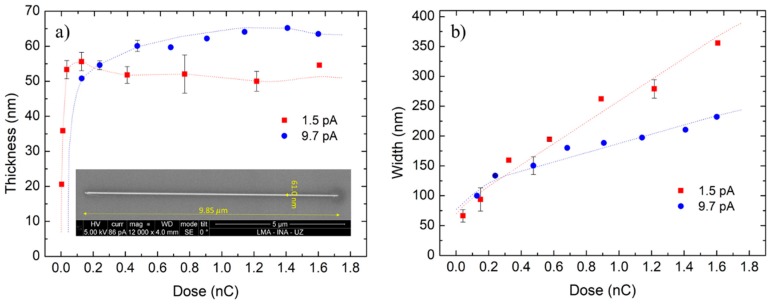
(**a**) Thickness and (**b**) width of Co-Focused Ion Beam Induced Deposition NWs (Co-FIBID NWs) as a function of the ion beam dose. The dimensions were directly determined while using the Dual Beam equipment measurement tools. The inset in (**a**) corresponds to a representative top-down SEM image of a Co-FIBID NW deposited with 1.5 pA, 0.017 nC.

As shown in Figure 1a, the NWs thickness abruptly increases at very low doses, up to a value where it remains approximately constant: between 48 nm and 58 nm for the current of 1.5 pA, and between 54 nm and 65 nm for the current of 9.7 pA. This result highlights the impossibility of growing these FIBID deposits with unlimited height. The competition between the material deposition and ion milling (something which is not present in FEBID) only enables obtaining these Co NWs with a thickness below ~65 nm. The subtle balance between the production of secondary electrons dissociating the precursor and the ion milling effect, which is different when the ion primarily impacts the Si substrate or the cobalt deposit itself can explain this effect. There is a point in which the growth and the ion milling compensate each other and thicker NWs cannot be grown under the available experimental conditions. A solution for this could be to increase the precursor flux to enhance the deposition rate, but this was not possible in the equipment used in this study, given that the gas injection valve was completely open during Co deposition and the GIS nozzle was already very close to the substrate.

In the case of the NWs width (Figure 1b), the tendency is not far from being linear. At low ion doses, the NWs are narrower for the low current of 1.5 pA (when considering the same ion dose), because the beam size is smaller. However, for higher ion doses, the NWs of 1.5 pA become wider than those of 9.7 pA. This occurs because low currents require several millions of passes (long deposit times of several minutes) to reach a high ion dose, which results in an important ion beam drift, leading to wider NWs.

Table 1 is a compilation of the most significant growth parameters that have been varied and the NWs dimensions achieved.

The calculated volume per dose reached values up to 0.31 µm^3^/nC for current 9.7 pA and up to 2.06 µm^3^/nC for current of 1.5 pA, largely overcoming the values that FEBID reached, typically around 0.002 µm^3^/nC [33]. As an example, the FIBID NW on Table 1 grown under 10 pA current for 48 s, with total volume of 0.0685 µm^3^, would have needed 57 min. if it was grown by FEBID with the same beam current. This indicates that the FIBID deposit fabrication can be at least more than 50 times faster than FEBID, which makes it a much more time-efficient technique. This great efficiency correlates with a typical production of several tens of secondary electrons per incident ion, which will be available for precursor dissociation. Previous work on the growth of cobalt nanowires by He^+^-FIBID suggests growth rates of around 1.0 µm^3^/nC [34].

According to the results in Table 1, it is remarkable how the growth rates rapidly reduce with dose, up to a value where the variation is not very significant. This considerable decrease is clearly linked to the ion milling present in FIBID, which increases with the ion dose. Thus, when considering the aim of getting high growth rates, as well as narrow and thick NWs, the optimal ion doses are those below 0.250 nC. According to Figure S1 in the Supplementary File, these NWs grown with ion doses in the optimal dose range are also those with less halo and more defined shape. The characterization of these optimized NWs will be shown in the next sections.

### 3.2. TEM

Cross-sectional lamellae of two Co-FIBID NWs grown with ion doses in the optimal range were examined: specifically, a NW deposited with ion current and dose 1.5 pA and 0.017 nC and another one grown with 9.7 pA and 0.224 nC, respectively. HRTEM imaging confirmed that (see Figure S2 in the Supplementary File) the deposits present the typical pseudo-amorphous structure that was also observed in Co-FEBID [38]. Both EELS and EDS in STEM analyzed the chemical composition.

#### 3.2.1. Compositional Analysis by STEM-EELS

**Figure 2:**
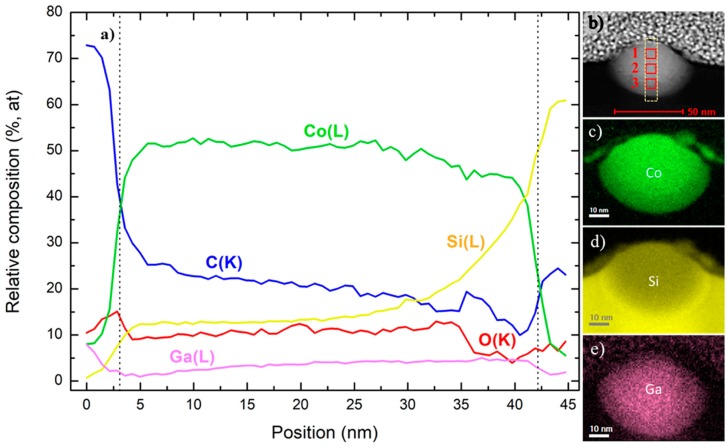
STEM-EELS analyses in the cross-section of NW 1.5 pA, 0.017 nC. (**a**) Vertical chemical profile of a 10-nm-wide rectangular region (light, dashed line) along the NW’s cross-section indicated in the HAADF-STEM image (**b**). The NW thickness is indicated by the dashed line in the profile. Two dimensional EELS areal density chemical maps of the significant elements (**c**) Co, (**d**) Si, and (**e**) Ga.

These STEM-EELS analyses provide valuable information regarding the spatial distribution of the chemical elements. According to Figure 2a, for the NW of 1.5 pA, 0.017 nC the Co content is around 50 at. % all along the cross-section, except in the area close to the substrate where it lowers.

The C percentage decreases along the NW thickness from 30 at. % near the surface to around 10 at. %, whereas the O goes from 10 at. % to 5 at. %. The Ga is uniformly distributed (Figure 2e) below 5 at. % and the Si tends to increase at the interface with the substrate, approximately in the same fashion as the Co and C decrease. The compositional maps (Figure 2c,d) highlight the intermixing between the NW and Si substrate, which is also suggested by the HAAD-STEM image (Figure 2b), showing a darker region (lower atomic number) in the bottom part than in the rest of the deposit.

**Figure 3:**
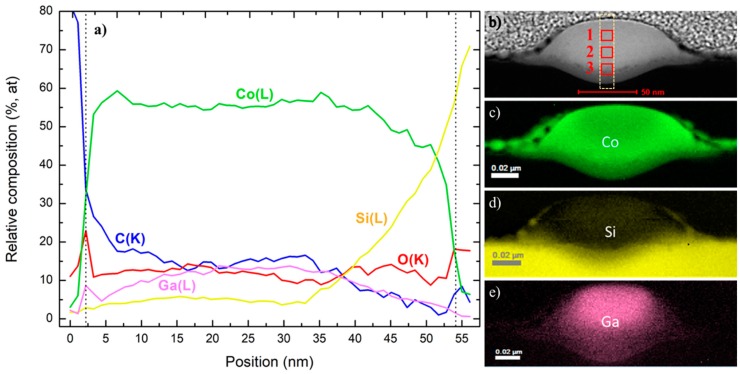
STEM-EELS analyses in the cross-section of NW 9.7 pA, 0.224 nC. (**a**) STEM-EELS vertical chemical profile of a 10-nm-wide rectangular region (light, dashed line) along the NW’s cross-section indicated in the HAADF-STEM image (**b**). The NW thickness is limited by the dashed line in the profile. 2D-EELS areal density chemical maps of the significant elements (**c**) Co, (**d**) Si, and (**e**) Ga.

As for the NW of 9.7 pA, 0.224 nC (Figure 3a), the Co content is between 55–60 at. %, decreasing when approaching the substrate, similarly to the previous NW. C also diminishes along the cross-section, whereas O seems to be more homogeneously distributed. The Ga content (near 15 at. %) is greater far from the substrate (as shown in Figure 3e) and higher than in the 1.5 pA, 0.017 nC NW. Finally, the Si considerably increases when reaching the substrate, again as a result of the intermixing of the substrate and deposit (Figure 3b–d), in a similar way than for the NW of 1.5 pA, 0.017 nC.

#### 3.2.2. Compositional Analysis by STEM-EDS

Three different regions (indicated with numbered red squares in Figure 2b and Figure 3b) were examined to quantify the chemical composition: one close to the surface (1), another one in the central part (2), and the last one near the substrate (3). Table 2 summarizes the results.

For the NW of 1.5 pA, 0.017 nC the Co content is reduced from 45.6 at. % in the top region to 24.6 at. % in the area close to the substrate. The same decreasing behavior is found for C and O. The Ga content remains constant at around 9 at. % through all the NW. Unlike the other elements, the Si percentage increases up to almost 50 at. % near the substrate, which indicates a high degree of chemical intermixing at the interface between the deposit and the substrate.

In the NW of 9.7 pA, 0.224 nC the Co content is around 50 at. % in the three regions. C, O, and also Ga diminish while approaching the substrate. Remarkably, the Ga percentage is very high at the surface, up to 26 at. %. Intermixing with Si at region 3 is important, becoming more significant when getting closer to the substrate.

In conclusion, the EELS and EDS results of the Co-FIBID NWs agree, with two key aspects to remark in comparison with Co-FEBID NWs: (1) the chemical intermixing of Co (C and O as well) with Si at the interface with the substrate and (2) the severe Ga doping. The intermixing with Si probably occurs at the early stages of growth, when the ion beam directly impinges on the substrate and it is caused by the high kinetic energy and momentum of Ga^+^ ions. Such Ga^+^ ions are also implanted in the NW, which is more significant for higher current and dose and close to the surface (up to 25 at. %). As a result, Ga doping is expected to play a crucial role in the transport and magnetic properties of the nanowires.

### 3.3. Analysis of the Transport Properties by Four-Point Electrical Microprobe Measurements

**Figure 4:**
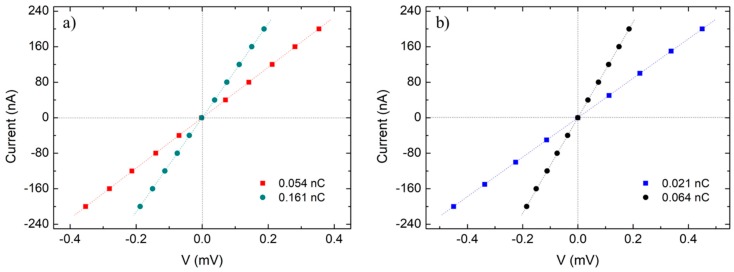
Electrical measurements (current versus voltage) of Co-FIBID NWs grown under (**a**) 1.5 pA and (**b**) 9.7 pA.

Room-temperature electrical measurements were carried out in Co-FIBID NWs that were deposited with different ion doses within the optimal range (<0.250 nC). Figure 4 shows representative results, where ohmic transport can be noticed. This is indicative of a metallic behavior arising from the percolation of the metallic cobalt + gallium grains.

For the NWs of 1.5 pA (Figure 4a), the resistivities are (37.7 ± 12.0) µΩ·cm for 0.054 nC and (25.6 ± 3.6) µΩ·cm for 0.161 nC. In the case of 9.7 pA, the results are (24.5 ± 3.5) µΩ·cm for 0.021 nC and (18.8 ± 4.0) µΩ·cm for 0.064 nC.

Such resistivity values are statistically equivalent, as they lie within the experimental error of the others, so a meaningful dependence of resistivity with the dose seems to be premature with the available data. These large statistical errors are probably due to uncontrolled variations in the morphology and the halo of NWs grown under the same conditions. In this sense, one of the largest sources of width variation has been found to be the beam drift.

If we compare with the existing literature on related materials, Co NWs grown by He^+^-FIBID exhibited resistivities comprised between 64 µΩ·cm and 116 µΩ·cm, as reported by Wu et al. [34]. Co-FEBID NWs that are grown on polycarbonate (PC) substrates displayed a resistivity of 30.5 µΩ·cm, as measured by Peinado et al. [39], whereas the found value was ~40 µΩ·cm when similar NWs are grown on Si-based substrates, as reported by Fernández-Pacheco et al. [33]. Co-FEBID deposits were found by Begun et al. to reach resistivity values below 25 µΩ·cm after performing post-growth purification treatments [29]. Similarly, after thermal annealing, Puydinger dos Santos et al. attained a resistivity of ~26 µΩ·cm [30]. We also note that the Co polycrystalline bulk resistivity has been reported to be 11 µΩ·cm [40] and the resistivity of Co electrodeposited films can be as low as 8.2 µΩ·cm [41].

**Figure 5:**
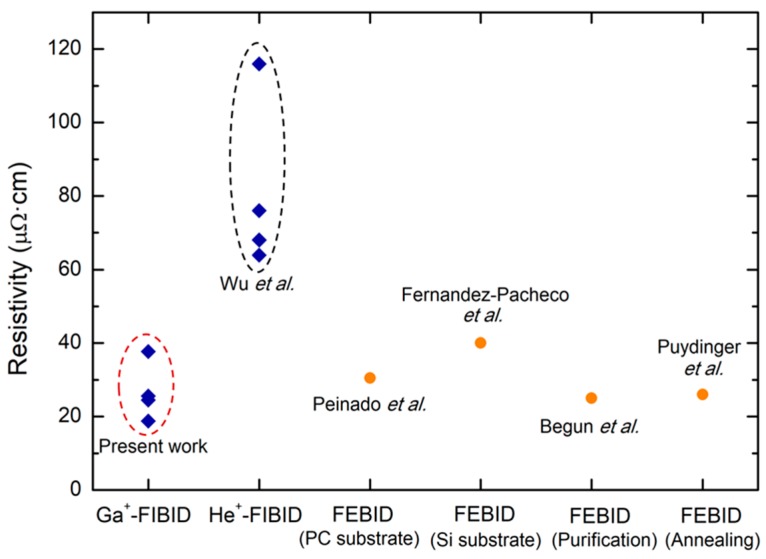
Comparison of the electrical resistivity values of Co-FIBID and Co-FEBID deposits grown under different conditions reported in the literature [29,30,33,34,39].

For the FEBID NWs, the resistivity decreases with the Co content, as expected. However, and according to the data shown in Figure 5, the Co NWs grown by Ga^+^-FIBID show resistivity values that are lower than those grown by He^+^-FIBID, and with comparable values to those of FEBID NWs. This is owing to the metallic Ga doping, which substantially improves the conductivity in spite of the lower Co content with respect to the FEBID NWs. Whereas the Ga contamination will be beneficial for applications exploiting the electrical conductivity of these NWs, it will be deleterious for applications that require high magnetization, as discussed in the next section.

### 3.4. Analysis of the Magnetization Hysteresis Loops by Magneto-Optical Kerr Effect

**Figure 6:**
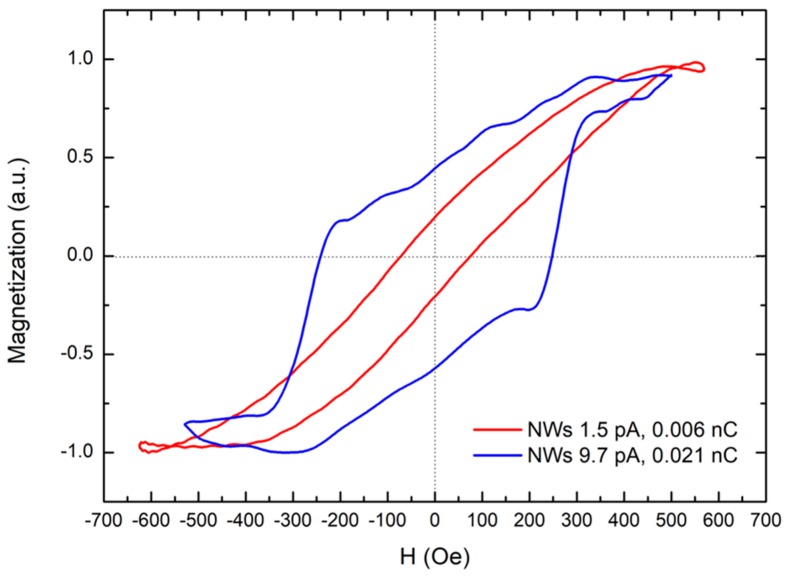
Hysteresis loops of Co-FIBID NWs with the magnetic field applied along the NW axis.

Figure 6 displays the MOKE loops of the Co-FIBID NWs, which were obtained after substraction of spurious high-field slopes and off-center compensation. The hysteretic behaviour proves the ferromagnetic nature of the NWs. Nevertheless, the loop shape is changing for NWs grown under different growth conditions, similarly to the coercive fields (H_C_): 72 Oe for 1.5 pA, 0.006 nC NWs and 251 Oe for 9.7 pA, 0.021 nC NWs. The different magnetic behaviour of the NWs can be explained by their shape [42], which has been explored, in detail, by SEM and TEM (see the Supplementary File). For the NWs grown under low current and ion dose, given their very small cross-sectional area, magnetization reversal through formation of magnetic domain walls and their propagation is unlikely. Instead, coherent rotation of the magnetization is more likely, whose signature is a small coercivity, as observed in Figure 6. The situation is different for the NWs that were grown under high current and ion dose. In this case, the NWs exhibit a higher coercive field and the hysteresis loop is unusual and it seems to be a combination of two contributions: (1) a first loop (narrow and similar to that of the 1.5 pA-NW) coming from the rounded, homogeneous part of the NW where the magnetization reversal is through coherent rotation; (2) a second loop (square-shaped, coming from the NW’s halo) where the magnetization reversal is through formation and the propagation of domain walls. The magnetic heterogeneity of high-dose NWs is likely to be a consequence of its irregular shape, which can be a limitation for those applications that require precise control of the magnetic stray field or the magnetization of the NWs. Moreover, the Ga contamination is expected to decrease the saturation magnetization of the NWs, which hampers applications that require high magnetization of the NWs.

## 4. Conclusions and Outlook

Co-FIBID NWs have been grown with a volume per dose beyond 50 times greater than their Co-FEBID counterparts. This feature is revealed as the greatest advantage of the FIBID technique with respect to FEBID for the growth of magnetic deposits. Moreover, Ga^+^-FIBID Co NWs have the advantage of a lower electrical resistivity, owing to the metallic doping introduced by the Ga^+^ implantation, with respect to He^+^-FIBID Co NWs. Such electrical resistivity values are amongst the lowest ones exhibited by any deposit grown by FEBID or FIBID, even after post-purification steps. In addition, it has been shown that Ga^+^-FIBID using low ion beam current (<10 pA) allows for the growth of Co NWs with lateral dimensions down to 50 nm. The structural and compositional characterization of the NWs revealed the relevance of Ga doping as well as the intermixing with the Si substrate. The ferromagnetic character of the Co NWs that were grown by Ga^+^-FIBID has been demonstrated, raising concerns about the magnetic homogeneity in the wider NWs, in contrast to the narrower ones. Further enhancement of the magnetic properties is expected after post-growth purification treatments, as previously demonstrated in Co-FEBID NWs. As a noticeable aspect, a limitation in thickness due to compensation between the material deposition and the ion milling has been found, which might be overcome by an increase of the local Co precursor flux on the growth area. One can speculate that a trade-off in the properties exhibited by Co NWs grown by FIBID could be achieved by using focused ion beam sources with atomic weight intermediate between that of Ga^+^ and He^+^. This could result in an intermediate lateral resolution within the range of 10 nm and 50 nm obtained, respectively, with He^+^ and Ga^+^ sources, and in intermediate resistivity values within the range of 20 and 120 µΩ·cm obtained, respectively, with Ga^+^ and He^+^ sources. In addition, the limitation in thickness that was observed due to Ga^+^ milling could be avoided with the use of lighter ion beams or with a higher precursor flux.

In summary, it has been shown that the growth of Co ferromagnetic nanostructures by Ga^+^-FIBID presents various advantages when compared to FEBID, with the two most important ones being the faster growth rate and the lower electrical resistivity. The best lateral resolution obtained by means of Ga^+^-FIBID has been found to be 50 nm. The observed limitation in the maximum deposit thickness, which is caused by the significant Ga^+^-induced milling, can be mitigated either by the use of higher precursor flux or by the use of lighter ion sources.

## Figures and Tables

**Table 1:** Growth parameters and dimensions (length x section) of Co-FIBID NWs. The section was estimated with ImageJ freeware software package. The current in the table corresponds to the experimental values (1.39 pA and 10.67 pA), instead of the nominal ones (1.5 pA and 9.7 pA).

Current (pA)	Deposit Time (s)	Dose (nC)	Dimensions	Volume Per Dose (µm^3^/nC)
1.39	3	0.004	9.78 µm × 879 nm^2^	2.062
1.39	12	0.017	9.85 µm × 2111 nm^2^	1.247
1.39	233	0.324	9.85 µm × 4282 nm^2^	0.130
1.39	466	0.648	9.84 µm × 5167 nm^2^	0.079
1.39	641	0.891	9.94 µm × 8507 nm^2^	0.095
1.39	926	1.287	9.94 µm × 9725 nm^2^	0.075
1.39	1156	1.607	10.1 µm × 13390 nm^2^	0.084
10.67	12	0.128	9.83 µm × 4037 nm^2^	0.310
10.67	21	0.224	9.75 µm × 5360 nm^2^	0.233
10.67	48	0.512	9.90 µm × 6922 nm^2^	0.134
10.67	64	0.683	9.94 µm × 8160 nm^2^	0.119
10.67	107	1.142	9.98 µm × 9322 nm^2^	0.081
10.67	132	1.408	9.78 µm × 9535 nm^2^	0.067
10.67	150	1.601	9.81 µm × 9758 nm^2^	0.060

**Table 2:** EDS composition (in atomic percentage) of Co-FIBID NWs of 1.5 pA, 0.017 nC and 9.7 pA, 0.224 nC.

1.5 pA, 0.017 nC	Element (Spectral Line) Atomic%		9.7 pA, 0.224 nC	Element (Spectral Line) Atomic%	
	C(K)	33.00 ± 0.90		C(K)	11.57 ± 0.33
	O(K)	9.89 ± 0.31		O(K)	14.34 ± 0.29
Region 1	Si(K)	2.43 ± 0.15	Region 1	Si(K)	0.28 ± 0.06
	Co(K)	45.59 ± 1.02		Co(K)	47.21 ± 0.80
	Ga(K)	9.07 ± 0.54		Ga(K)	26.57 ± 0.75
	C(K)	22.14 ± 0.59		C(K)	15.31 ± 0.40
	O(K)	8.93 ± 0.28		O(K)	11.45 ± 0.28
Region 2	Si(K)	18.62 ± 0.35	Region 2	Si(K)	2.88 ± 0.12
	Co(K)	41.83 ± 0.90		Co(K)	52.63 ± 0.83
	Ga(K)	8.45 ± 0.48		Ga(K)	17.71 ± 0.59
	C(K)	14.94 ± 0.62		C(K)	6.66 ± 0.37
	O(K)	3.44 ± 0.26		O(K)	8.78 ± 0.30
Region 3	Si(K)	48.32 ± 0.67	Region 3	Si(K)	24.47 ± 0.45
	Co(K)	24.61 ± 0.80		Co(K)	48.88 ± 1.06
	Ga(K)	8.66 ± 0.63		Ga(K)	11.19 ± 0.64

## Supplementary Materials

The following are available online at https://www.mdpi.com/2079-4991/9/12/1715/s1, 1. Morphological analysis by SEM imaging; 2. HRTEM imaging.
