# Supplementary material for: High Volume-Per-Dose and Low Resistivity of Cobalt Nanowires Grown by Ga+ Focused Ion Beam Induced Deposition
Source: Nanomaterials (Basel). 2019 Dec 1;9(12):1715. doi: 10.3390/nano9121715 (PMC6955673; doi:10.3390/nano9121715)
Supplement: Supplementary file 1 [file nanomaterials-09-01715-s001.pdf]

nanomaterials

MDPI

Article

High volume-per-dose and low resistivity of cobalt nanowires grown by Ga^+ Focused Ion Beam Induced Deposition

Carlos Sanz-Martín [1], César Magén [1,2,3] and José María de Teresa [1,2,3],*

[1] Instituto de Ciencia de Materiales de Aragón (ICMA), Universidad de Zaragoza-CSIC, 50009 Zaragoza, Spain.
[2] Departamento de Física de la Materia Condensada, Universidad de Zaragoza, 50009 Zaragoza, Spain.
[3] Laboratorio de Microscopías Avanzadas (LMA), Instituto de Nanociencia de Aragón (INA), Universidad de Zaragoza, 50018 Zaragoza, Spain.
* Correspondence: deteresa@unizar.es

Received: date; Accepted: date; Published: date

1. Morphological analysis by SEM imaging

The cross-section of Co-FIBID NWs is analyzed in Figure S1. Qualitative chemical information is also provided by the secondary electron SEM images, since heavier elements (Co of the NW and Pt of the deposit coverage) emit more secondary electrons than lighter ones (Si of the substrate, in this case). Regarding the deposits shape, for small ion doses, the NWs have quite well-defined rounded section. However, as the dose increases, the NW cross-sectional shape tends to be more spread and wider each time (and with an increasing halo), though the thickness does not experience significant changes.

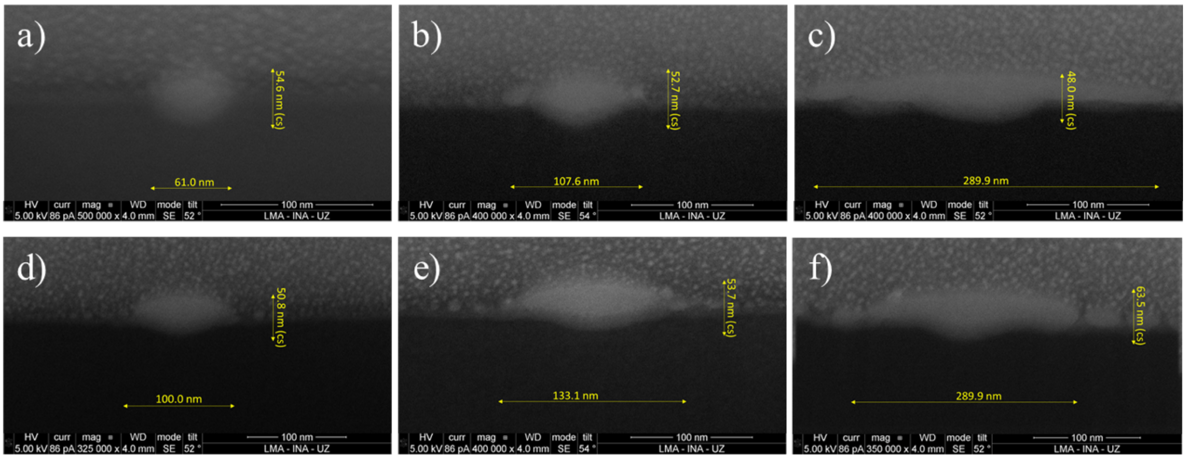

Figure S1. SEM images of Co-FIBID cross-sections for current 1.5 pA with dose (**a**) 0.017 nC, (**b**) 0.161 nC, (**c**) 1.287 nC; and current 9.7 pA with dose (**d**) 0.128 nC, (**e**) 0.224 nC and (**f**) 1.601 nC.

2. HRTEM imaging

HRTEM images (Figure S2) display a nanocrystalline microstructure, with no texture. The Fast Fourier Transform (FFT) reveals just a diffuse ring, indicating the lack of defined atomic planes and, thus, the presence of a pseudo-amorphous structure. In these HRTEM images, lower-density elements (Si as substrate) appear brighter than the heavier species (Co from the NW and Pt from the

protective layer for the TEM lamella preparation). It is possible to appreciate how the NW region closer to the substrate seems to have an average density lower than the rest of the deposit, suggesting a chemical intermixing with the Si. In addition, these HRTEM images confirm that the NWs shape is more defined for lower ion doses, whereas it tends to spread as the dose increases.

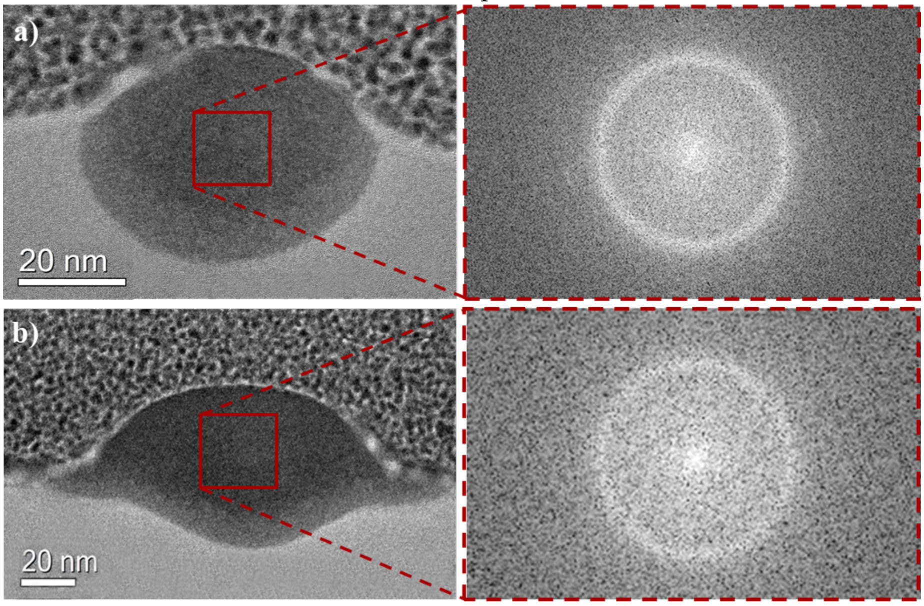

Figure S2. HRTEM images of Co-FIBID NWs of (**a**) 1.5 pA, 0.017 nC and (**b**) 9.7 pA, 0.224 nC. Each HRTEM image is accompanied by the corresponding FFT of the indicated area of the image.

© 2019 by the authors. Submitted for possible open access publication under the terms and conditions of the Creative Commons Attribution (CC BY) license (http://creativecommons.org/licenses/by/4.0/).
